# Nano-sized mesoporous phosphated tin oxide as an efficient solid acid catalyst†

**DOI:** 10.1039/c8ra08962k

**Published:** 2019-01-08

**Authors:** S. M. Hassan, M. A. Mannaa, Amr Awad Ibrahim

**Affiliations:** Chemistry Department, Faculty of Science, Mansoura University Mansoura Egypt amr_awad@mans.edu.eg; Chemistry Department, Faculty of Education and Science, Amran University Sa'dah Yemen; Department of Chemistry, Virginia Commonwealth University Richmond VA 23284-2006 USA

## Abstract

Herein, we prepared a mesoporous tin oxide catalyst (mSnO_2_) activated with phosphate species by the adsorption of phosphate ions from a phosphoric acid solution onto tin oxyhydroxide (Sn(OH)_4_) surface. The phosphate content ranged from 3 to 45 wt%. The nonaqueous titration of *n*-butylamine in acetonitrile was used to determine the total surface acidity level. FTIR of chemically adsorbed pyridine was used to differentiate between the Lewis and Brönsted acid sites. Thermal and X-ray diffraction analysis indicated that the addition of phosphate groups stabilized the mesostructure of mSnO_2_ and enabled it to keep its crystalline size at the nanoscale. FTIR analysis indicated the polymerization of the HPO_4_^2−^ groups into P_2_O_7_^4−^, which in turn reacts with SnO_2_ to form a SnP_2_O_7_ layer, which stabilizes the mesoporous structure of SnO_2_. The acidity measurements showed that the phosphate species are distributed homogeneously over the mSnO_2_ surface until surface saturation coverage at 25 wt% PO_4_^3−^, at which point the acid strength and surface acidity level are maximized. The catalytic activity was tested for the synthesis of hydroquinone diacetate, where it was found that the % yield of hydroquinone diacetate compound increased gradually with the increase in PO_4_^3−^ loading on mSnO_2_ until it reached a maximum value of 93.2% for the 25% PO_4_^3−^/mSnO_2_ catalyst with 100% selectivity and excellent reusability for three consecutive runs with no loss in activity.

## Abstract 2

Herein, we prepared a mesoporous tin oxide catalyst (mSnO_2_) activated with phosphate species by the adsorption of phosphate ions from a phosphoric acid solution onto tin oxyhydroxide (Sn(OH)_4_) surface.

## Graphical abstract

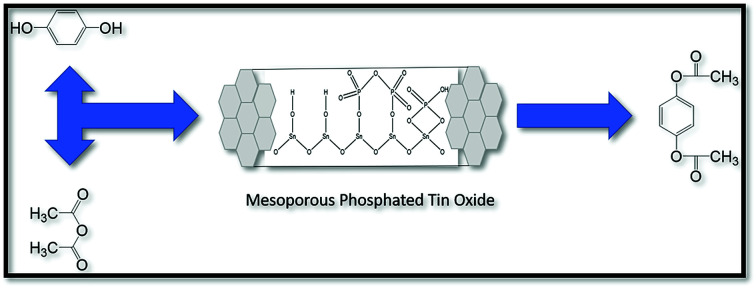

## Introduction

The replacement of liquid acid or acid halide catalysts, such as H_2_SO_4_, H_3_PO_4_, HF and AlCl_3_, is a critical factor for the design of cleaner processes for green catalytic action.^1^ The problems of homogeneous liquid acid catalysis include toxicity, corrosion, catalyst waste and difficulty in separation and recovery.^2^ The economics of the separation cost majorly determine the viability of such a process.^3^ Efforts have been made to design environmentally safe processes and technologies to achieve these goals. The best catalytic technology for solving the problems of the liquid acid catalyst is to replace it by a heterogeneous solid acid catalyst.^4^ The reuse and recycling of the catalyst in heterogeneous processes are the main advantages of the increased catalyst efficiency over homogeneous ones.^5^ The presence of an interconnected network of a large number of pores, acid sites of different strength and the hydrophobic properties of solid catalyst surfaces support inhibiting the processes of corrosion and separation and for limiting pollution problems.^2^ Oil refining and pharmaceutical industries utilize solid catalysts with acidic properties.^6–8^ SO_4_^2−^, PO_4_^3−^, WO_4_^2−^, Cl^−^ and F^−^ supported on metal oxides have been used to increase their thermal stability, mesoporosity, and more critically, the acidity level of such catalysts.^9–12^ Most of the published works to date have been focused on SO_4_^2−^- and WO_4_^2−^-supported metal oxide systems,^13–18^ which have been found to be suitable for specific acid-catalyzed reactions. The acidity of such systems strongly depend on the preparation method.^11,14,16^

A template pathway for preparing mesoporous materials is a novel process to produce materials with high activity for the synthesis of industrially relevant chemicals.^19–22^ Mesoporous materials are characterized by a high surface area, ordered pore channels, and large pore volume value. These advantages can facilitate active components achieving a highly dispersed state on the support, which raises their activity for reacting molecules.^23,24^ For better performance, an efficient heterogeneous catalyst needs a higher acid strength level and density as well as thermal stability to minimize catalyst poisoning and leaching. Therefore, mesoporous materials, primarily metal oxides, have attracted growing interest as a support in the field of catalysis.^25^ Tin oxide is one of the most attractive functional materials because of its potential applications, mainly as a catalyst and as a carrier in supported catalysts.^26^ Besides its intrinsic non-stoichiometry and crystal defects, tin oxide nanoparticles can be used more efficiently as a catalyst due to the faster migration of the oxide ions within the SnO_2_ nanoparticles to increase the surface to volume ratio. Many authors have used high valence cations of metal oxide to promote the thermal stability and catalytic property of pure tin oxide.^27,28^ Therefore, it is of interest to study the novelties of phosphate-supported SnO_2_ as a solid acid catalyst. Various chemical routes have been used to synthesize nanoparticles of mesoporous metal oxides, *e.g.*, precipitation, sol–gel, hydrothermal, microwave-assisted syntheses and ultrasonic spray pyrolysis.^29,30^ The addition of anion groups to a mesostructure metal oxide during the preparation methods prevent the sintering process and thus lead to improvements in their thermal stability and textural properties.^31,32^

Further improvement is necessary, however, for the preparation of thermally stable mesoporous tin oxide. This process is still a challenge to researchers.^9,33^ Unlike sulfated metal oxides, phosphated metal oxides have received inadequate attention. Albeit, phosphate-based solid acids have proven quite efficacious in several industrially important acid-demanding reactions.

The present study aimed to adopt a phosphate-modified mesoporous tin oxide (mSnO_2_) system as a catalyst. The modified catalytic systems (H_3_PO_4_/m-SnO_2_) were then applied to the preparation of hydroquinone diacetate, which is widely used as an ingredient for skin bleaching cream. DTA was used to systematically elucidate the physico-chemical properties of the H_3_PO_4_/mSnO_2_ catalysts, while TGA, XRD, TEM and FTIR techniques were applied for characterization, and the texture properties were determined from N_2_ adsorption at −196 °C. The surface acidity was also examined.

## Results and discussion

**Fig. 1:**
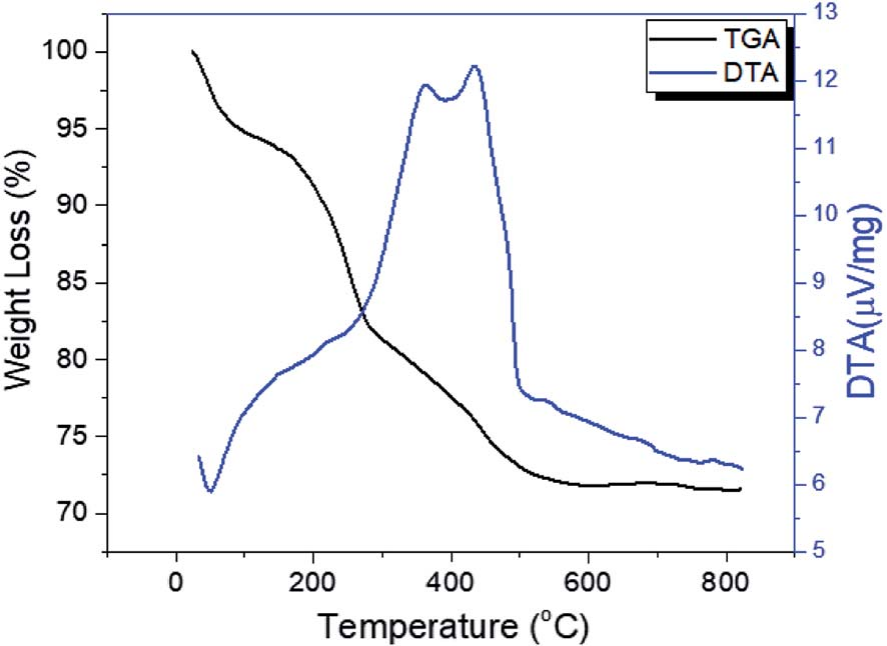
DTA and TGA curves of dried Sn(OH)_4_.

**Fig. 2:**
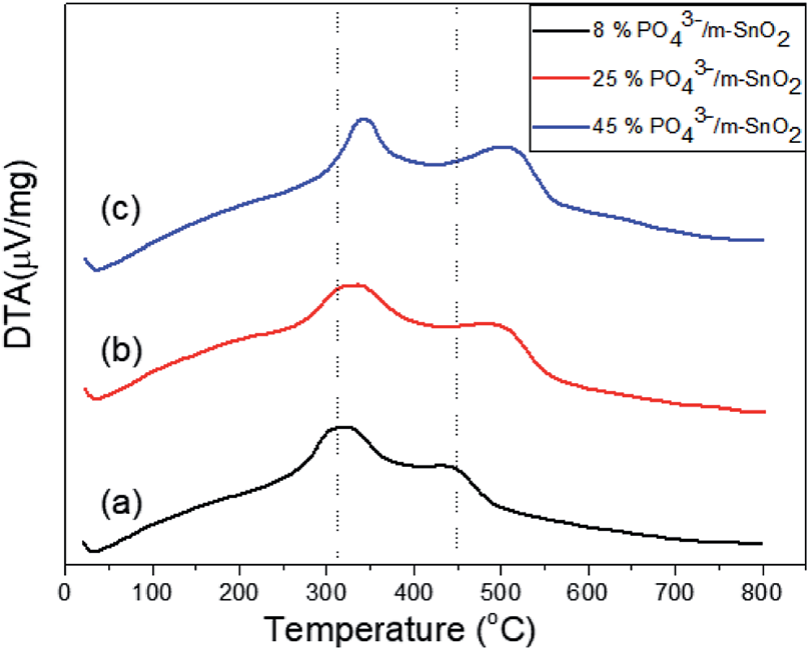
DTA curves of (a) 8%, (b) 25%, (c) 45% PO_4_^3−^/m-SnO_2_ samples.

The thermograms of DTA and the TGA of dried PO_4_^3−^/Sn(OH)_4_ samples are shown in Fig. 1. The sample showed endothermic peaks at low temperature, which are assigned to the evolution of physically adsorbed water. The sample also showed two exothermic peaks. The first exothermic effect with a weight loss of 8.9 wt% at around 312 °C was attributed to the decomposition of the surfactant template and partial removal of the OH groups (dehydroxylation),^34,35^ while the second exothermic peak at 423 °C corresponded to a weight loss of 3.1 wt%, which may be due to the crystallization of tin oxyhydroxide into tetragonal cassiterite SnO_2_.^36^ In the case of PO_4_^3−^/Sn(OH)_4_ dried samples, the DTA in Fig. 2 shows exothermic peaks at 321 °C, 334 °C and 342 °C as the phosphate content increases for the 8%, 25% and 45% PO_4_^3−^/Sn(OH)_4_ samples, respectively. Also, the exothermic peaks of the crystallization of tin oxyhydroxide into mSnO_2_ were also shifted to higher temperatures as the phosphate content increased, reaching 427 °C, 484 °C and 504 °C for the 8%, 25% and 45% PO_4_^3−^/mSnO_2_ samples, respectively, indicating that the addition of phosphate to mSnO_2_ may enhance the surfactant–tin oxyhydroxide surface interaction,^37^ thus increasing the thermal stability of the tin oxyhydroxide phase and shifting its crystallization to higher temperature. These results show no effect that could be assigned to the decomposition of PO_4_^3−^ species, which was attributed to the thermal stability when treated with phosphoric acid.^38^

Fig. 2S(A)† shows the low-angle XRD patterns of 3%, 25%, and 35% PO_4_^3−^/mSnO_2_ samples heated at 400 °C. The XRD curves exhibit one diffraction peak at 2*θ* = 2.4°, corresponding to the (100) reflection, which was assigned to the presence of hexagonal mesostructured SnO_2_.^39,40^ The same peak was observed for the calcined samples at 400 °C, 550 °C and 650 °C (Fig. 2S†), which indicates that the presence of phosphate groups maintain the mesoporous structure of mSnO_2_.

**Fig. 3:**
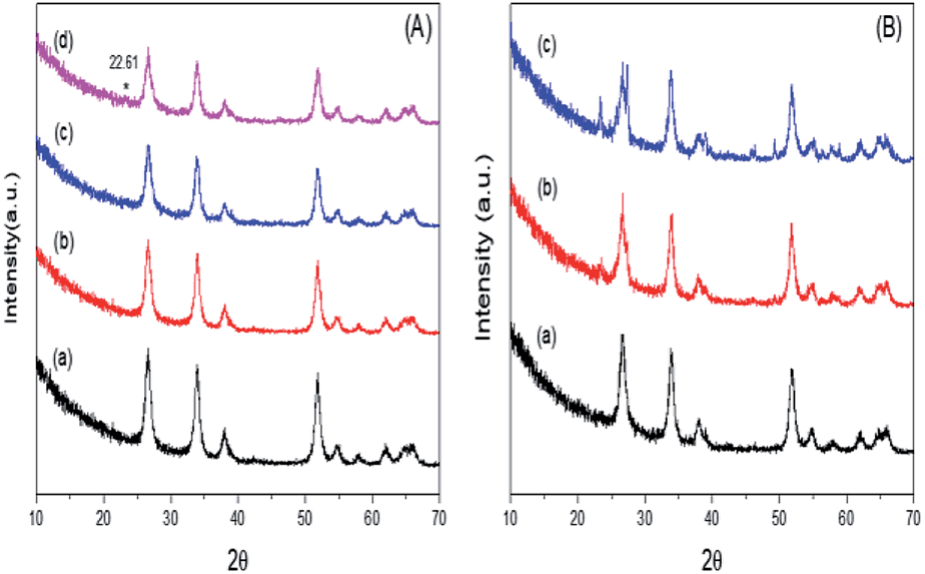
(A) Wide-angle XRD pattern of (a) m-SnO_2_ (400 °C) and the samples of PO_4_^3−^/m-SnO_2_ (400 °C) at (b) 3%, (c) 25%, (d) 35%, (B) wide-angle XRD patterns of the sample 3% PO_4_^3−^/m-SnO_2_ at (a) 400 °C, (b) 550 °C, (c) 650 °C.

Fig. 3A illustrates the wide-angle XRD patterns of mSnO_2_, and for the 3%, 25% and 35% PO_4_^3−^/mSnO_2_ calcined at 400 °C. The samples were found to display four well-distinguished peaks at 2*θ* = 26.64°, 33.91°, 38.10° and 51.74°, corresponding to the (110), (100), (200) and (211) reflection planes of a tetragonal cassiterite phase of mSnO_2_, respectively. The addition of PO_4_^3−^ to mSnO_2_ until 25 wt% PO_4_^3−^ shows an XRD pattern similar to that of the pure mSnO_2_, indicating that the PO_4_^3−^ species exists as amorphous phosphate groups on the mSnO_2_ surface.^41^ When the loading of PO_4_^3−^ increased to 35 wt%, a new reflection peak appears at 2*θ* = 22.61°, which can be assigned to a layer-structured SnP_2_O_7_.^42^ On the other hand, it is clear from Fig. 3B that the intensity of the diffraction peaks of the 3% PO_4_^3−^/mSnO_2_ sample increases with the increase in the calcination temperature of the sample from 400 °C to 650 °C, which may be due to the increase in the dispersion of PO_4_^3−^ on the mSnO_2_ surface, which in turn increase the thermal stability of mSnO_2_, arising from the interaction of PO_4_^3−^ and mSnO_2_. The degree of crystallization of cassiterite mSnO_2_ gradually decreases with the increase in the phosphate content, while the width of the reflections is considerably broadened, indicating a small crystalline domain size (Table 1). The addition of phosphate anions to the template synthesis reaction medium of mSn(HO)_4_, substantially slows down the crystallization process *via* retarding the crystal size growth by occupying the defects on the surface of mSn(HO)_4_,^43^ thus increasing its thermal stability and thus protecting the mesoporous structure from collapsing.^41^ The crystallite size data were determined from X-ray diffraction and are listed in Table 1, showing that the crystallite size of PO_4_^3−^/mSnO_2_ is smaller in comparison to the pure mSnO_2_ nanocrystalline (13.70–11.30 nm). Furthermore, the crystallite size of 3% PO_4_^3−^/mSnO_2_ increases from 12.17 to 16.66 nm with the increase in calcination temperature (400–650 °C), which indicates that pure mSnO_2_ and PO_4_^3−^/mSnO_2_ are present in the nanoscale. In the phosphate sample annealed between 400 °C and 650 °C, the crystallite size slightly changes from 13.70 to 16.66 nm, suggesting that the PO_4_ species remain bonded to the mSnO_2_ surface, stabilizing it against sintering. It was reported that the order of the mesostructured materials strongly depends on the texture properties of the pores.^44^ An analogous phenomenon was observed with TiO_2_, ZrO_2_ and Fe_2_O_3_.^44,45^

**Table 1:** Comparison of the structural parameters of mSnO_2_ and various PO_4_^3−^/mSnO_2_ samples

Samples	*D* (nm)	*d*_100_ (nm)	*d*_pore,BJH_ (nm)	*a* ^0^ (nm)	*t*_wall_ (nm)
mSnO_2_-400	13.70	3.62	3.22	4.18	0.96
3%PO_4_^3−^/mSnO_2_-400	12.17	3.63	3.15	4.19	1.04
25%PO_4_^3−^/mSnO_2_-400	11.87	3.66	2.66	4.23	1.57
35%PO_4_^3−^/mSnO_2_-400	11.30	3.69	2.52	4.26	1.74
3%PO_4_^3−^/mSnO_2_-550	14.50	3.60	3.41	4.16	0.75
3%PO_4_^3−^/mSnO_2_-650	16.66	3.57	3.82	4.13	0.31

**Fig. 4:**
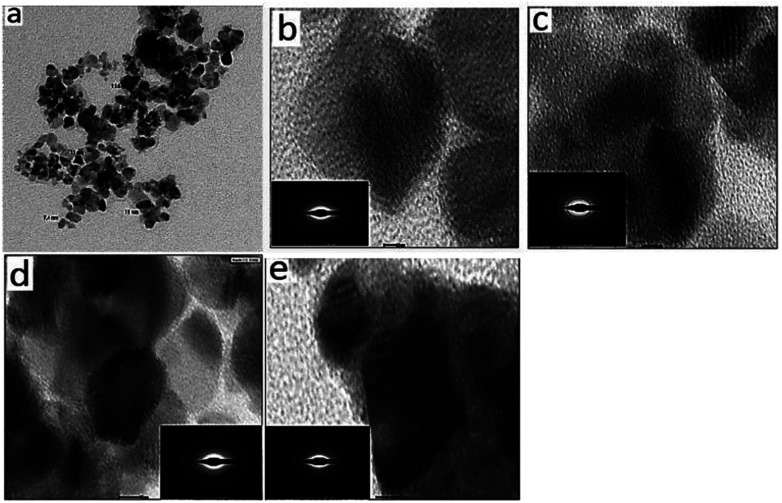
TEM images of (a) mSnO_2_ calcined at 550 °C, (b) 3% PO_4_^3−^/m-SnO_2_ calcined at 400 °C, (c) 3% PO_4_^3−^/m-SnO_2_ calcined at 550 °C, (d) 8% PO_4_^3−^/m-SnO_2_ calcined at 400 °C and (e) 25% PO_4_^3−^/m-SnO_2_ calcined at 400 °C.

TEM images of mSnO_2_ show an ordered structure with a uniform mesoporous arrangement into hexagons (Fig. 4a). It is apparent that mSnO_2_ has a significant void fraction, which may be due to the mesoporous structure of SnO_2_, and concomitantly a rather low density. Fig. 4c–e show the TEM images of the 3%, 8% and 25% PO_4_^3−^/mSnO_2_ catalysts, which indicate that when PO_4_^3−^ species are loaded on mSnO_2_ calcined at 400 °C, the lattice retains the ordered mesoporous channels and the PO_4_^3−^ groups are embedded uniformly into the mSnO_2_ framework without disordering the mesostructured framework. The selected area electron diffraction (SAED) pattern images (insets) of the samples exhibit diffraction rings attributed to the polycrystalline behaviour of the mSnO_2_, while the crystallite degrees of the 3%, 8% and 25% PO_4_^3−^/mSnO_2_ catalysts decrease with the increase in PO_4_^3−^ content.^46,47^ The TEM images of 3% PO_4_^3−^/mSnO_2_ calcined at 550 °C (Fig. 4b) illustrate that the mesoporous structure remains even after calcination at 550 °C, which reflects the role of the phosphate groups in improving the thermal stability of the tin oxide.^47^ It is clear from the TEM images that the dark colour and the area occupied by the phosphate group both increase with both the phosphate content and the calcination temperature, indicating the great dispersion of phosphate groups on the mesoporous SnO_2_ surfaces. Also, the side images (inset) show an increase in the crystalline size with increasing calcination temperature, which is in a good agreement with the results from the XRD analysis.

**Fig. 5:**
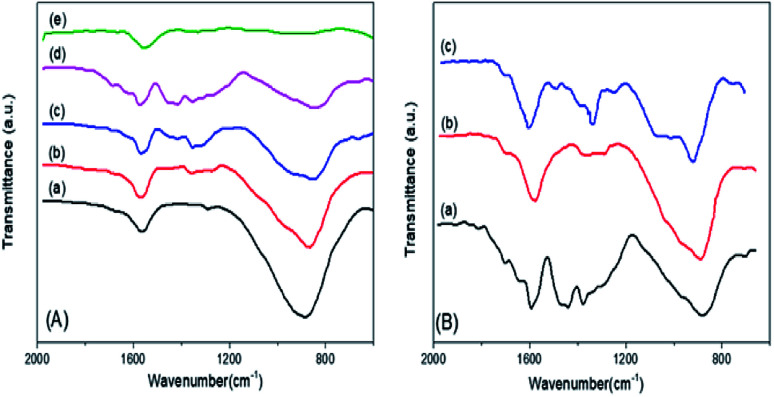
(A) FTIR spectra of (a) m-SnO_2_ (550 °C) and (b) 3%, (c) 25%, (d) 35%, (e) 45% PO_4_^3−^/m-SnO_2_ catalysts calcined at 550 °C. (B) FTIR spectra of 3% PO_4_^3−^/m-SnO_2_ calcined at: (a) 450 °C, (b) 550 °C, (c) 650 °C.

The FTIR spectra of the mSnO_2_ 550 °C, *x*PO_4_^3−^/mSnO_2_ and 3PO_4_^3−^/mSnO_2_ samples calcined at different temperatures are shown in Fig. 5A and B. The band at 1625 cm^−1^ appearing in the spectra of the samples even after calcination at different temperatures corresponds to the OH bending vibrations. The FTIR spectra of phosphate mSnO_2_ generally show absorption bands at 1035 and 1150, 1360, 1410 and 1520 cm^−1^. The absorption bands at 1360, 1410 and 1520 cm^−1^ could be assigned to P–O stretching frequency bonds in which their order is close to two P=O, phosphoryl groups.^48^ By increasing the temperature to 650 °C, the broadband at 1550–1300 cm^−1^ is reduced to one sharp band and one shoulder, due to the formation of bidentate-bound phosphate ions (C_2_V point group). The band at 1035 and the shoulder at 1150 cm^−1^ are frequencies for PO_4_^3−^ and the position of such a fundamental band was observed in phosphated zirconia.^49^ The fading of the shoulder and the presence of a broad and sharp band located at 1055 cm^−1^ when the PO_4_^3−^ content was increased to 45 wt% may be due to the dropping of the symmetry in the free PO_4_^3−^ (Td point group) to either C_3_V or C_2_V.^48^ This may be due to the PO_4_^3−^ groups being bound to the tin oxide surface and being distributed homogeneously.^50,51^ Here, the 1055 cm^−1^ band is divided into two bands at 1035 and 1150 cm^−1^ at 650 °C, which could be assigned to the bidentate bound phosphate ions (C_2_V point group), which motivate the polymerization of the HPO_4_^2−^ groups into P_2_O_7_^4−^, which is in a good agreement with the appearance of a new peak due to SnP_2_O_7_ in the X-ray pattern (Fig. 3A).

**Scheme 1:**
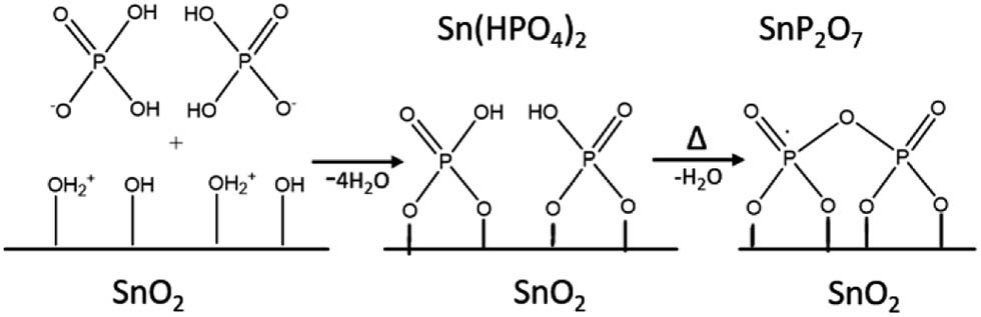
Formation of PO_4_^3−^/mSnO_2_.

These results indicate that phosphate groups are chemically adsorbed on the surface of mSnO_2_ and still exist on the surface even after calcination of the samples at high temperatures. The mechanism, as explained in Scheme 1, follows the dehydroxylation reaction of P/mSnO_2_ samples, with amorphous Sn(HPO_4_)_2_ formed on the surface, which is further heated to form tin pyrophosphate, SnP_2_O_7_, which is in a good agreement with the XRD pattern at 650 °C.

**Fig. 6:**
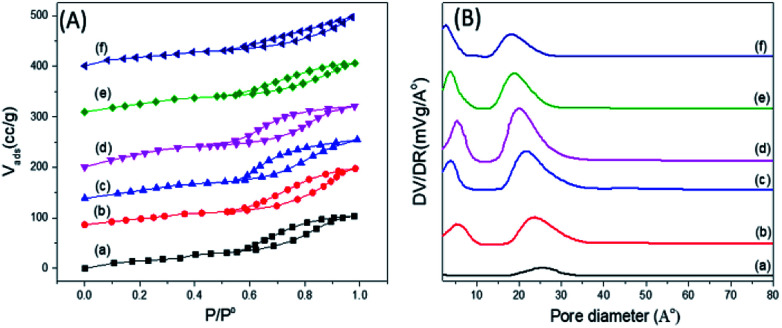
(A) Adsorption–desorption isotherms of nitrogen at −196 °C (B) pore size distributions for PO_4_^3−^/m-SnO_2_ at (a) 3%, (b) 8%, (c) 18%, (d) 25%, (e) 35%, (f) 45% calcined at 400 °C.

Fig. 6 shows the nitrogen adsorption–desorption isotherms of some representative samples of PO_4_^3−^/mSnO_2_, in which the isotherms exhibit a type IV feature, which is a characteristic feature of mesoporous materials.^52^ Barrett–Joyner–Halenda (BJH) analysis demonstrated that the average pore diameter (*d*_pore,BJH_) of mSnO_2_ calcined at 550 °C was 13.21 nm (Table 1). The *d*_pore,BJH_ of PO_4_^3−^/mSnO_2_ samples was found to decrease with the increase in phosphate content from 3.15 to 2.52 nm (Table 1). The approximate thickness of the porous wall (*t*_wall_) calculated from the difference between the hexagonal unit cell parameter (*a*^0^ = 2*d*_100_/√3) and *d*_pore,BJH_ (Table 1), after calcination at 400 °C *t*_wall_ increased with increasing the phosphate content from 0.96 nm for mSnO_2_ to 1.74 nm for the 35% PO_4_^3−^/mSnO_2_ samples. The *d*_pore,BJH_ and *t*_wall_ values decreased with the calcination temperature. On the other hand, the values of *d*_110_-spacing and *a*^0^ were found to decrease with the increase in calcination temperature, which may indicate that some changes occur on the mesostructure as the calcination temperature of PO_4_^3−^/mSnO_2_ increases. The values of *S*_BET_, as shown in Table 2, increase gradually with the rise of PO_4_^3−^ content to reach a maximum for 25% PO_4_^3−^/mSnO_2_. This amount was optimal to form a monolayer, and the values of *S*_BET_ decreased with further increases of the PO_4_^3−^ content.

**Table 2:** Surface characteristics of PO_4_^3−^/m-SnO_2_ catalysts

Catalyst	*T* (°C)	*S*_BET_ m^2^ g^−1^	*d*_pore,BJH_ (nm)	VT ml g^−1^
m-SnO_2_	550	29	3.22	0.13
3% PO_4_^3−^/m-SnO_2_	400	65	3.15	0.16
8% PO_4_^3−^/m-SnO_2_	400	95	2.96	0.17
18% PO_4_^3−^/m-SnO_2_	400	130	2.81	0.18
25% PO_4_^3−^/m-SnO_2_	400	155	2.66	0.18
35% PO_4_^3−^/m-SnO_2_	400	113	2.52	0.14
45% PO_4_^3−^/m-SnO_2_	400	75	2.46	0.14
3% PO_4_^3−^/m-SnO_2_	450	63	3.35	0.15
3% PO_4_^3−^/m-SnO_2_	550	56	3.41	0.15
3% PO_4_^3−^/m-SnO_2_	650	41	3.82	0.14

Moreover, the *S*_BET_ values were found to decrease when the calcination temperature increased up to 650 °C.^50,51^ The increase in the surface area values of PO_4_^3−^/mSnO_2_ over that of mSnO_2_ may be due to the formation of two-dimensional surface layers from Sn-PO_4_^3−^, which stabilize the defects and inhibit the collapsing of the mSnO_2_ surface,^53^ which also explains the role of PO_4_^3−^ in enhancing the porous structure of PO_4_^3−^/mSnO_2_. The increase in PO_4_^3−^ content beyond 25 wt% led to the production of more phosphate layers and/or the phosphate groups attacking the pore mouths blocking them, and consequently the surface area values decreased.^54,55^ The above results suggest that the interaction between PO_4_^3−^ and mSnO_2_ crystallites not only stabilizes the crystallite structure in lower dimensions, but also it keeps phosphate groups at the SnO_2_ surfaces, inhibiting the crystallite aggregation and acting as a structure progeny director mediating nanoparticle growth and assembly and stabilizing the mesostructure walls and increasing the thermal stability.^56^ Again, the increase in surface area and pore volume and the decrease in the average pore diameter of the 3% to 25% PO_4_^3−^/mSnO_2_ samples over pure mSnO_2_ with increasing the phosphate content indicate a relatively high thermal stability of the prepared samples. The decrease in the pore volume and *d*_pore,BJH_ upon increasing the phosphate content over 25 wt% may be due to the formation of multi-phosphate layers, as proposed in Scheme 1, whereby the surface hydroxyl groups of Sn(OH)_4_ are replaced by phosphate groups and therefore there is an increased energy barrier and less crystal growth sites, consequently inhibiting the crystal growth.

The samples showed a bimodal pore size distribution consisting of smaller and larger interparticle pores, as illustrated in Fig. 6B. The addition of PO_4_^3−^ to mSnO_2_ was associated with an increase in the number of mesopores and micropores, as evident from the increase in the height of the distribution maxima and slight shift to lower rH values until a maximum at 25 wt% phosphate was reached, where it begins to decrease. Upon increasing the calcination temperature, (Fig. 3S†), both the smaller and larger interparticle pores decreased, which is in line with the decrease in the surface (Table 2). The width of the mesopore type increased with phosphate content, which confirmed the role of PO_4_^3−^ in increasing the porosity of mSnO_2_.^56^

The nonaqueous potentiometric titration curves of *n*-butylamine of Fig. 4S† were used to calculate the total number of acid sites and the acid strength of the PO_4_^3−^/mSnO_2_ catalysts.^57,58^ As evident from Fig. 4S† and the data in Table 3, the acidity and the acid strength values of PO_4_^3−^/mSnO_2_ changed with the phosphate loading and reached their maximum value for 25% PO_4_^3−^/mSnO_2_ (Ei = +480.6 mV) calcined at 400 °C, which may be due to the completion of a bidentate phosphate monolayer on the tin oxide surface, as evident from the FTIR data. The decrease in the total acidity and the acid strength upon increasing the PO_4_^3−^ loading may be due to the formation of more phosphate layers on the mSnO_2_ surface. The calcination temperature 400 °C was the most suitable calcination temperature for the generation of strong acidic sites.

**Table 3:** Acidity characteristics of PO_4_^3−^/m-SnO_2_ catalysts

Catalyst	*T* (°C)	Ei (mV)	No. of acid sites/g × 10^−19^	B/L	Conversion%	TOF h^−1^
m-SnO_2_	400	74	0.84	0.54	0	0
3% PO_4_^3−^/m-SnO_2_	400	320.4	0.96	1.04	8.6	222.88
8% PO_4_^3−^/m-SnO_2_	400	372.6	1.08	0.82	50.3	1158.76
18% PO_4_^3−^/m-SnO_2_	400	450.7	1.20	1.12	87.7	1818.31
25% PO_4_^3−^/m-SnO_2_	400	480.6	1.33	1.68	93.2	1743.47
35% PO_4_^3−^/m-SnO_2_	400	421.8	0.96	1.14	80.4	2083.70
45% PO_4_^3−^/m-SnO_2_	400	382.3	0.72	1.48	63.1	2180.45
25% PO_4_^3−^/m-SnO_2_	450	295.6	0.90	1.07	85.4	2360.83
25% PO_4_^3−^/m-SnO_2_	550	274.6	0.84	1.52	61.6	1824.53
25% PO_4_^3−^/m-SnO_2_	650	203.5	0.78	1.36	30.5	972.87

The loss of surface acidity at higher calcination temperatures up to 650 °C may be due to the loss of H^+^ by dehydration with the surface OH or OH of the next phosphate groups, forming a bridging phosphate group on the surface, as shown in Scheme 1, which causes a reduction in acidic hydrogen and a decrease in the total acidity.

**Fig. 7:**
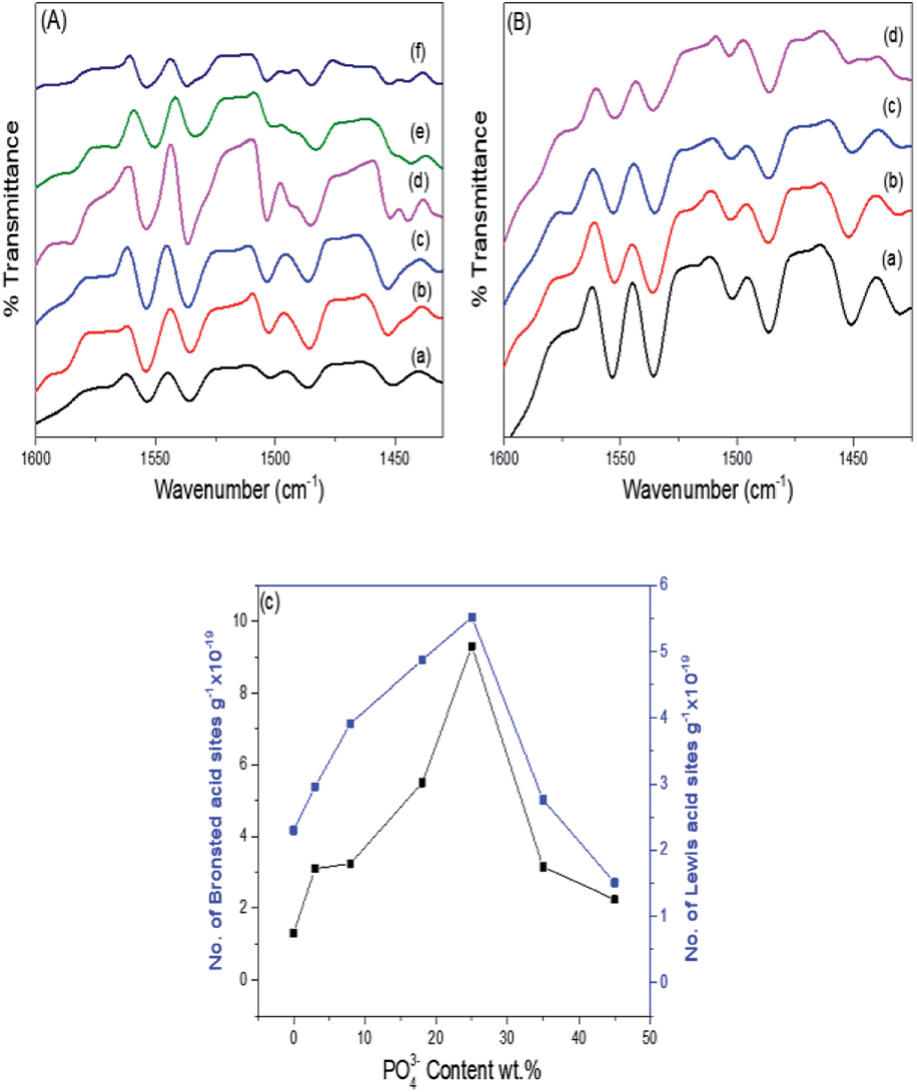
(A) FTIR spectra of pyridine adsorbed on (a) 3%, (b) 8%, (c) 18%, (d) 25%, (e) 35%, (f) 45% PO_4_^3−^/m-SnO_2_ calcined at 400 °C. (B) FTIR spectra of pyridine adsorbed on the 25% PO_4_^3−^/m-SnO_2_ sample calcined at (a) 400 °C, (b) 450 °C, (c) 550 °C and (d) 650 °C. (C) Effect of PO_4_^3−^ content on the number of Brönsted and Lewis acid sites.

**Scheme 2:**
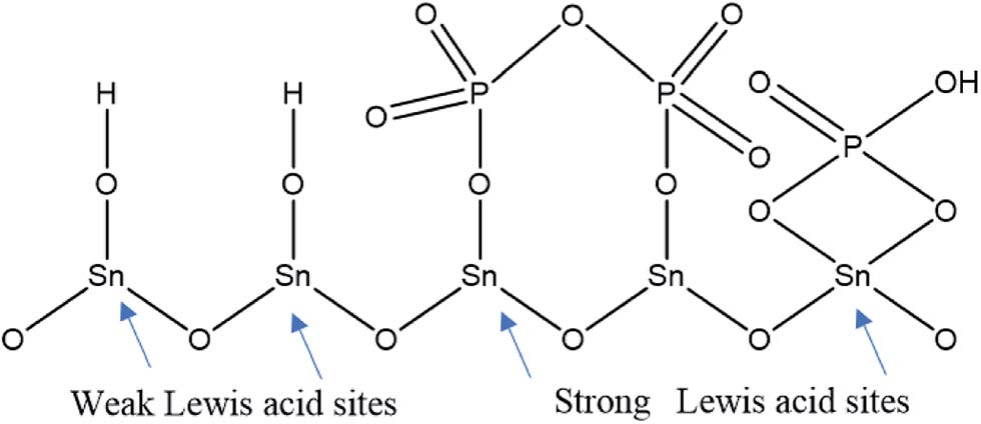
Lewis acid on PO_4_^3−^ /mSnO_2_.

Pyridine adsorption on the PO_4_^3−^/mSnO_2_ samples was used to specify the Brönsted (B) and Lewis (L) acid sites on the sample surfaces. The FTIR spectra of pyridine adsorbed over the Brönsted (B) and Lewis (L) acid sites of the samples are shown in Fig. 7A and B. Bands at around 1444 and 1452 cm^−1^ were characteristic of pyridine adsorbed on Lewis acid sites.^59^ The bands at 1536 and 1553 cm^−1^ were due to the pyridinium ion adsorbed on Brönsted acid sites.^60^ Moreover, other FTIR bands appear at 1484 and 1503 cm^−1^, attributed to the overlap of Lewis and Brönsted acid sites.^61^ The integrated areas of the bands at 1536 and 1452 cm^−1^ were used to calculate the number of Brönsted and Lewis acid sites. It was evident from Fig. 7C that the samples have Brönsted and Lewis acid sites and these increase with the increase in phosphate loading up to 25% PO_4_^3−^/mSnO_2_, and then decrease with further increases in PO_4_^3−^ loading above 25 wt%. Moreover, the number of Brönsted and Lewis acid sites decreases gradually with the increase in calcination temperature over 400 °C (Table 3). The surface distribution and interaction of phosphate species with the SnO_2_ surface increase the number of Brönsted and Lewis acid sites. In analogy with sulfated tin oxide,^62^ the increase in Lewis acidity by phosphate loading may be due to the formation of a tin oxide surface phosphorus complex containing P=O covalent bonds, and so the inductive effect of these covalent bonds of P=O strengthen the Lewis acid character of the sample, as suggested in Scheme 2.^63,64^ Fig. 5S† shows the NH_3_-TPD results for the pure m-SnO_2_ calcined at 400 °C, which shows a broad peak of NH_3_ desorption at 240 °C, which indicates the presence of weak acid sites. After modifying the mSnO_2_ with PO_4_^3−^, this peak starts to disappear and two peaks start to appear at 334 °C and 440 °C, corresponding to moderate and strong acid sites, respectively. For 45% PO_4_^3−^, only one peak at 440 °C is seen, corresponding to the strong acid sites due to the saturation of the surface with the PO_4_^3−^ groups.^26^

**Fig. 8:**
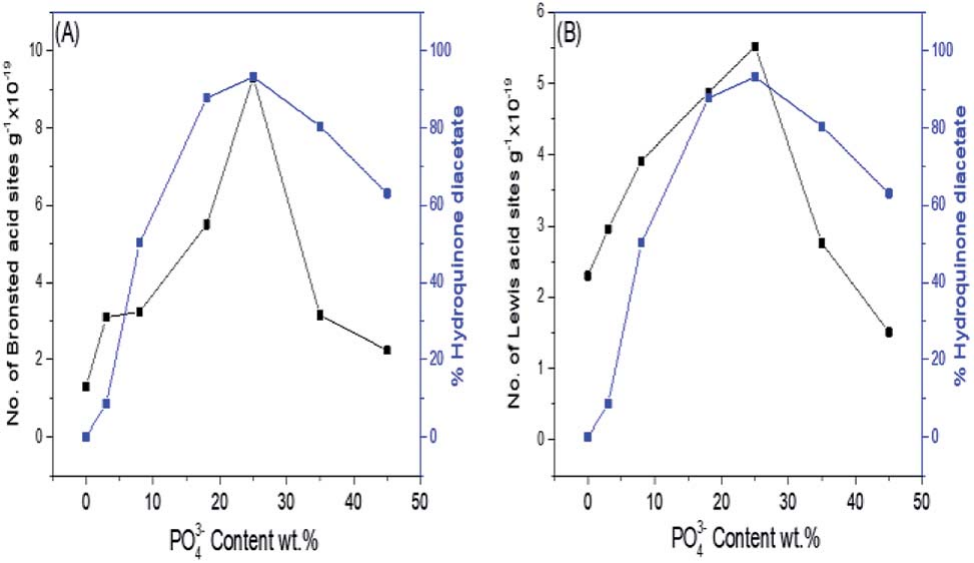
(A) Effect of PO_4_^3−^ content wt%/mSnO_2_ calcined at 400 °C on the number of Brönsted acid sites and % hydroquinone diacetate. (B) Effect of PO_4_^3−^ content wt%/mSnO_2_ calcined at 400 °C on the number of Lewis acid sites and % hydroquinone diacetate.

Fig. 8A and B show the effect of the phosphate content and consequently, the number of Brönsted and Lewis acid sites on the % formation of hydroquinone diacetate compound. As can be seen, the % yield of hydroquinone diacetate compound increases gradually with the increase in PO_4_^3−^ loading on mSnO_2_ until it reaches a maximum value of 93.2% for the 25% PO_4_^3−^/mSnO_2_ catalyst with a selectivity of 100%. The surface acidity and acid strength of PO_4_^3−^/mSnO_2_ are enhanced and attain a maximum at 25 wt% PO_4_^3−^. Moreover, the Brönsted and Lewis acid sites change with the same trend as the formation of hydroquinone diacetate compound, which may indicate that Lewis and/or Brönsted acid sites can catalyse the formation of hydroquinone diacetate compound. No activity was observed for pure mSnO_2_, which may be due to its low acidity and site strength (+74 mV). As evident from Table 3 and Scheme 2, increasing the calcination temperature from 400 °C to 650 °C lead to a decrease in the catalytic activity, which is in line with the decrease in surface area and the decrease of the number of Brönsted and Lewis acid sites. The calcination temperature is an essential aspect in the synthesis of phosphated metal oxide catalyst due to its role in the interaction between the metal oxide surface and phosphate groups for the generation of active acid sites. The optimum operating conditions for an anion-metal oxide catalyst was found to depend on the type of metal oxides, supported anions and the calcination temperature.^52^

It was observed that with increasing the molar ratio from 1 : 1 to 1 : 2 and 1 : 3, the % yield of hydroquinone diacetate increased gently from 60.2%, to 77.5% to 93.2%, respectively, with 100% selectivity. However, a further increase in the acetic anhydride molar content was accompanied by a decrease in the percentage yield of hydroquinone diacetate to 83.1% (molar ratio 1 : 4). As acetic anhydride acts as both a solvent and reactant, an excess of acetic anhydride is used to facilitate the reaction until the molar ratio of hydroquinone to acetic anhydride is 1 : 3. Many factors may affect the catalyst activity; the dilution effect of acetic anhydride (solvent effect) and/or the catalyst active sites may be blocked due to a higher acetic anhydride concentration, thereby decreasing the yield of hydroquinone diacetate. Because the activation of acetic anhydride is the key step in the formation of hydroquinone diacetate, the decrease in % yield of hydroquinone diacetate may be due to the presence of low (molar ratio 1 : 1 and 1 : 2) or excess (molar ratio 1 : 4) activated acetic anhydride – Brönsted acid sites and/or activated acetic anhydride – Lewis acid sites intermediates. Therefore, 1 : 3 molar ratio of reactants was considered optimum for the further studies.

**Fig. 9:**
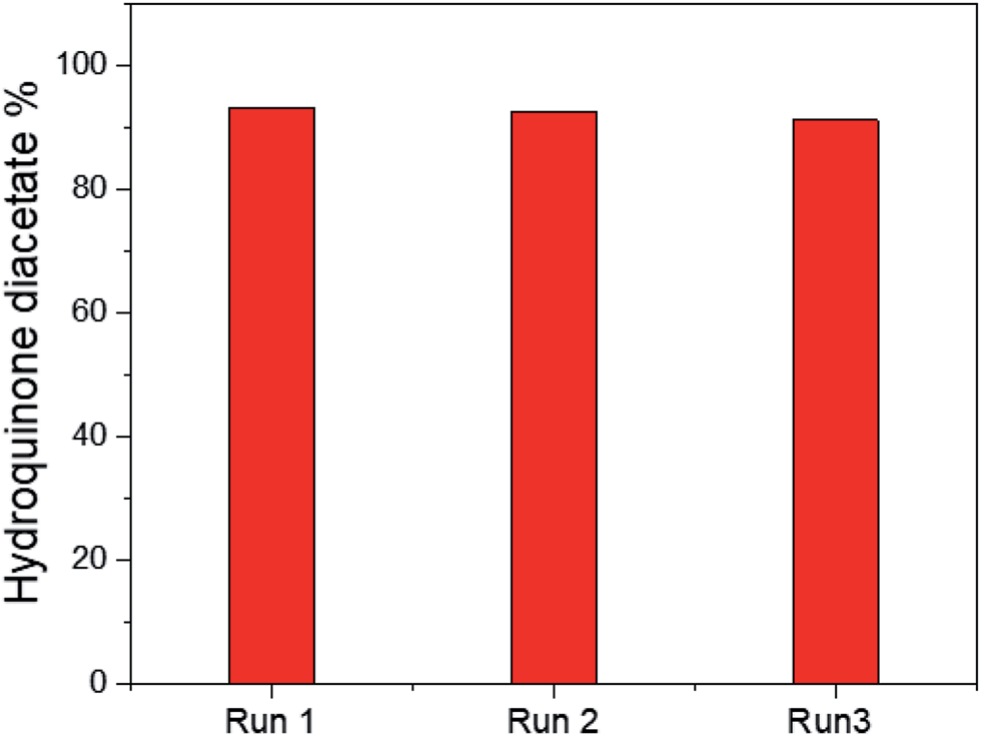
Reusability test for 25% PO_4_^3−^/mSnO_2_.

The reusability of the 25% PO_4_^3−^/mSnO_2_ catalyst was checked with three consecutive experiments by using the recovered catalyst. The catalyst was separated by filtration, washed with acetone several times and dried overnight at 100 °C. The reaction was reperformed on this reactivated catalyst. It was observed that there was no significant loss in activity, as shown in Fig. 9, and the catalytic activity of the catalyst was 93.2%, 92.4% and 91.1%, respectively. Moreover Fig. 7SA and B† show the TEM images of the 25% PO_4_^3−^/mSnO_2_ after the third run, which showed no change in the morphology of the reused catalyst; also the FTIR of the pyridine adsorbed on the reused sample showed no significant loss in the acidity. The obtained results indicated that the catalyst had excellent reusability over three consecutive runs.

### Reaction mechanism

**Scheme 3:**
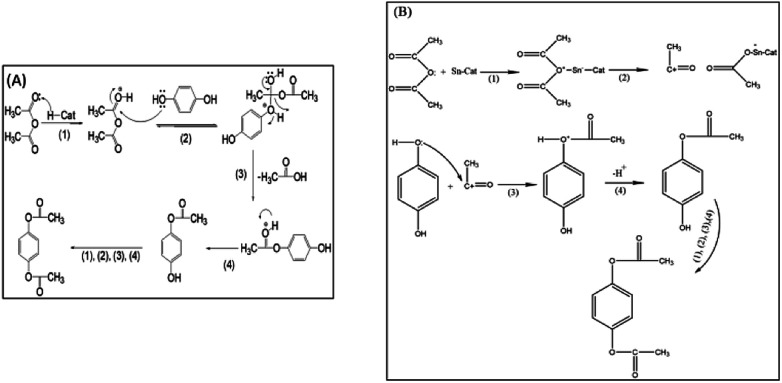
Reaction mechanism for the formation of hydroquinone diacetate on: (A) Brönsted acid sites of a solid acid catalyst, (B) Lewis acid sites of a solid acid catalyst.

The first step in the formation of hydroquinone diacetate is chemisorption of the carbonyl group of acetic anhydride on the Brönsted acid sites or Lewis acid sites of the catalyst, then on the adsorbed acetic anhydride on the Brönsted acid sites, Scheme 3A shows a nucleophilic attack by OH of hydroquinone to form an intermediate that breaks down to lose acetic acid in the next step. Finally, the proton of the Brönsted acid site is eliminated to give hydroquinone acetate. The hydroquinone acetate reacts with another acetic anhydride – Brönsted acid site to give the final product. The mechanism of the formation of hydroquinone diacetate on Lewis acid sites starts by the chemisorption of the carbonyl group on the acetic anhydride, which breaks down to form acylium ion. Then the OH of hydroquinone attacks acylium ion to form an intermediate, which lose a proton to give hydroquinone acetate, which undergoes the same steps to give the product hydroquinone diacetate, Scheme 3B.

## Experimental

### Synthesis of PO_4_^3−^/mSnO_2_

For the synthesis of mesoporous tin oxide (mSnO_2_), 15 ml of 25 wt% NH_4_OH solution was added to 100 ml solution containing 2.0 g CTAB dropwise and stirred for 15 min until the solution became homogeneous. 4.6 g SnCl_4_·5H_2_O was dissolved in 100 ml of distilled water and then added to NH_4_OH CTAB solution slowly.^65,66^ The pH was fixed at 8.5. The product was filtered and washed with 2 wt% CH_3_COONa solution until excess surfactant, and Cl^−^ ions were removed. The sample was then dried overnight at 800 °C.

A series of 3, 8, 18, 25, 35, and 45 wt% PO_4_^3−^-supported mSnO_2_ samples were prepared by suspending 3.0 g of dried mSnO_2_ in 25 ml of distilled water; then the calculated amount of 1 M H_3_PO_4_ solution was added dropwise to the suspension during stirring for 2 h. Then the samples were calcined at 400 °C, 450 °C, 550 °C and 650 °C for 4 h. 3, 8, 18, 25, 35 and 45 are denoted for the wt% of PO_4_^3−^.

### Characterization

DTA and TGA of the uncalcined samples were carried out using a Shimadzu thermal analyzer, type 50-H. The measurements were carried out under N_2_ flow of 20 ml min^−1^ with a heating rate of 10 °C min^−1^. The XRD patterns were recorded using a PW 150 (Philips). The crystallite size (nm), unit cell *d*_110_ and *a*^0^ were calculated from the strongest peak 110 of mSnO_2_ at 2*θ* = 26.54° by Bragg's law.^67^*nλ* = 2*d* sin(*θ*)*a*° = 2*d*/√3

TEM images of the calcined PO_4_^3−^/mSnO_2_ samples were obtained using a Joel JEM-1230 operated at 120 KV. FTIR spectra of the calcined samples were recorded by using MATTSON 5000 FTIR spectrophotometer (4 cm^−1^ resolution and 16 scans) in dried KBr (Merck). Physically adsorbed nitrogen at −196 °C was used for elucidation of the texture properties of the calcined P/mSnO_2_ samples. The total acidity of the solid samples was measured by suspending 0.05 g of solid catalyst in 10 ml of acetonitrile for 3 h and titrating with 0.1 N *n*-butylamine base in acetonitrile.^35,57^ An Orion 420 digital A pH-meter was used for measuring the potential variation. Lewis and Brönsted acid levels were determined from the exposure of the degassed solid samples at 200 °C to dried pyridine.^68^ After the removal of excess pyridine, a MATTSON 5000 FTIR spectrophotometer was used to record the spectra. For the NH_3_-TPD experiment, 200 mg of the samples were kept under ammonia gas for 4 h at 100 °C. Then the samples were flushed with helium, and then the temperature was raised from 50 °C to 700 °C at a ramping rate of 5 °C min^−1^ in the presence of helium, and the amount of NH_3_ desorbed was recorded using a TCD detector.

### Catalytic activity

The synthesis of hydroquinone diacetate (1,4-diacetoxybenzene) was used to test the catalytic activity of the samples. 0.1 g of the activated catalyst at 120 °C was added to 3 ml (0.032 mol) of acetic anhydride and 1.1 g (0.01 mol) of hydroquinone in a 50 ml flask, and the mixture then stirred for 15 min. After 15 min, the clear solution was filtered to separate the catalyst, and the filtrate was poured into 500 ml of crushed ice. The crystalline solids were dried to constant weight. The product was identified by FTIR, NMR spectroscopy and melting point determination. The % yield of hydroquinone diacetate was then calculated.

## Conclusions

Mesoporous SnO_2_ was successfully prepared through a simple method and loaded with different amounts of phosphate species. The XRD and TEM images showed that the samples were mesoporous. Phosphate species were homogeneously adsorbed on the surface of mesoporous SnO_2_ up to surface saturation coverage at 25 wt% PO_4_^3−^ at which point the surface, area surface acidity level and acid strength were at a maximum. The acidity of the samples was found to decrease when the phosphate loading exceeded 25 wt%, at which point polyphosphate multilayers were formed. Both Lewis and/or Brönsted acid sites were present on the catalyst surface. The synthesis of hydroquinone diacetate was used as a model reaction for testing the activity of the solid sample for the formation of bulky molecules. The maximum formation of hydroquinone diacetate and the surface acidities were found at 25 wt% phosphate loading and 400 °C calcination temperature, respectively. The catalyst could be successfully used many times without significant loss of its catalytic activity.

## Conflicts of interest

There are no conflicts to declare.

## Supplementary Material

RA-009-C8RA08962K-s001
