# Supplementary material for: Nano-sized mesoporous phosphated tin oxide as an efficient solid acid catalyst
Source: RSC Adv. 2019 Jan 8;9(2):810–8. doi: 10.1039/c8ra08962k (PMC9059524; doi:10.1039/c8ra08962k)
Supplement: RA-009-C8RA08962K-s001 [file RA-009-C8RA08962K-s001.pdf]

Electronic Supplementary Material (ESI) for RSC Advances.
This journal is © The Royal Society of Chemistry 2018

Nanosized mesoporous phosphated tin oxide as efficient solid acid catalyst

S. M. Hassan[a], and M. A. Mannaa[a,b], Amr Awad Ibrahim[a,c*]

[a] Chemistry Department, Faculty of Science, Mansoura University, Mansoura, Egypt

[b] Chemistry Department, Faculty of Education and Science, Amran University, Sa'dah Yemen.

[C] Department of Chemistry, Virginia Commonwealth University, Richmond, VA 23284-2006, United States

[a,c*] Corresponding authors., amr_awad@mans.edu.eg, aamohammed@vcu.edu

Supporting information

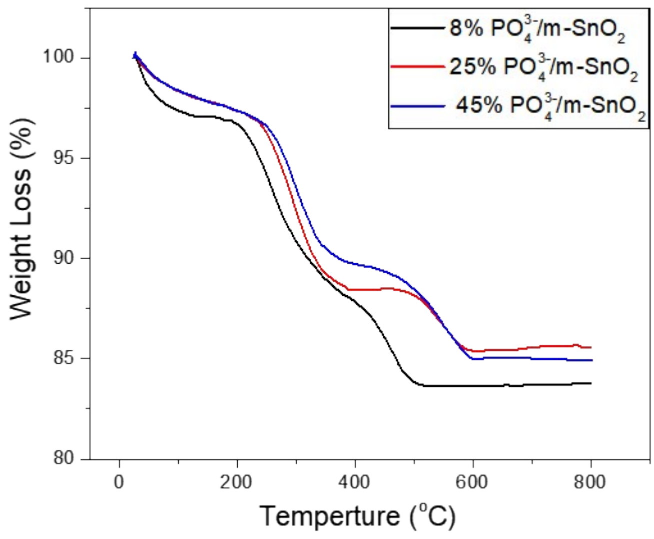

Fig. 1S: TGA curves of (a) 8%, (b) 25% (c) 45% PO_4^{3-}/m-SnO_2 samples.

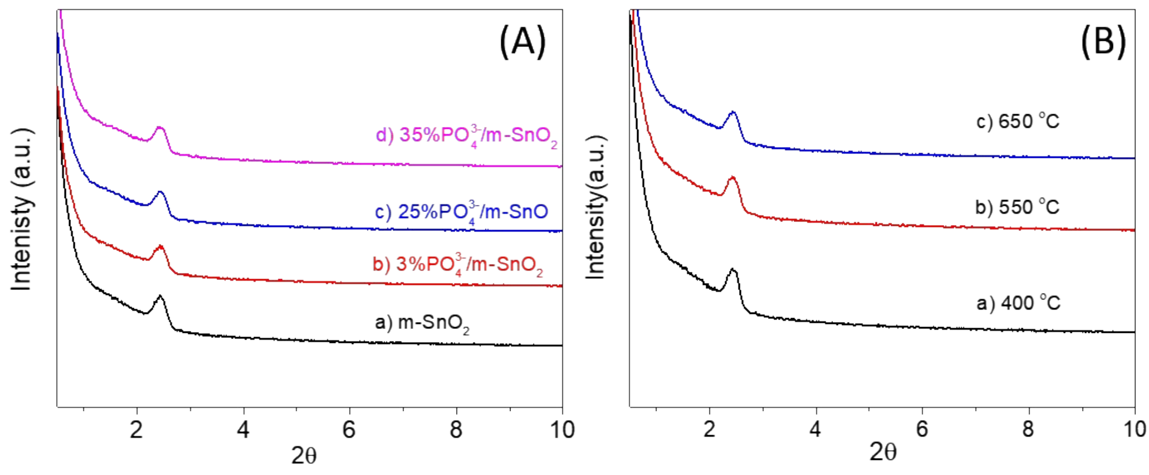

Fig.2S: (A)Low angle XRD pattern of the mesoporous (a) m-SnO_2 (550°C) and the samples PO_4^{3-}/SnO_2 (400°C) at (a) m-SnO_2 (b) 3% (c) 25% (d) 35%, (B) Low angle XRD pattern of the sample 3PO_4^{3-}/SnO_2 at different temperatures (a) 400°C (b) 550°C (c) 650°C.

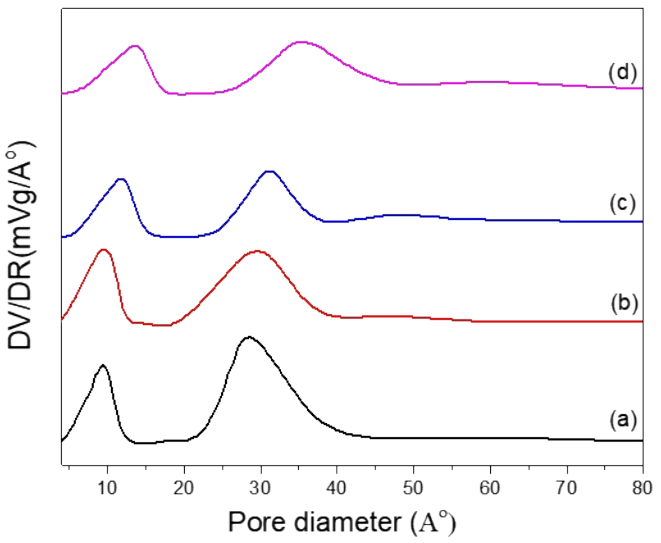

Fig.3S: Pore size distributions of 25% PO_4^{3-}/m-SnO_2 samples at (a) 400°; (b) 450°; (c) 550°; (d) 650°C

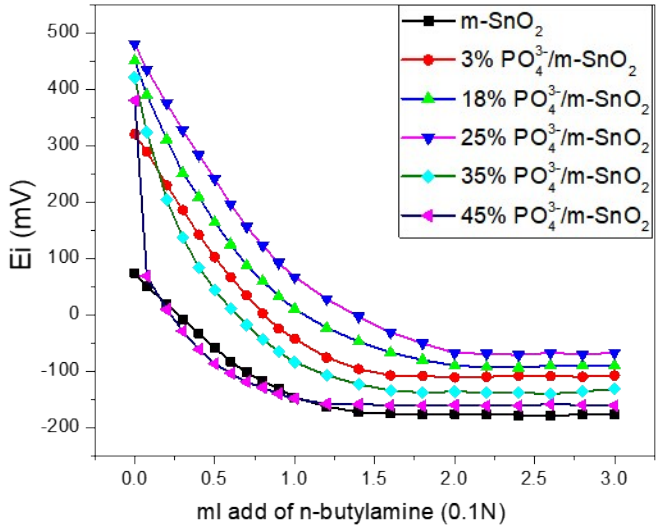

Figure 4S: Potentiometric titration of n-butylamine in acetonitrile for PO_4^{3-}/m-SnO_2 catalysts calcined at 400°C.

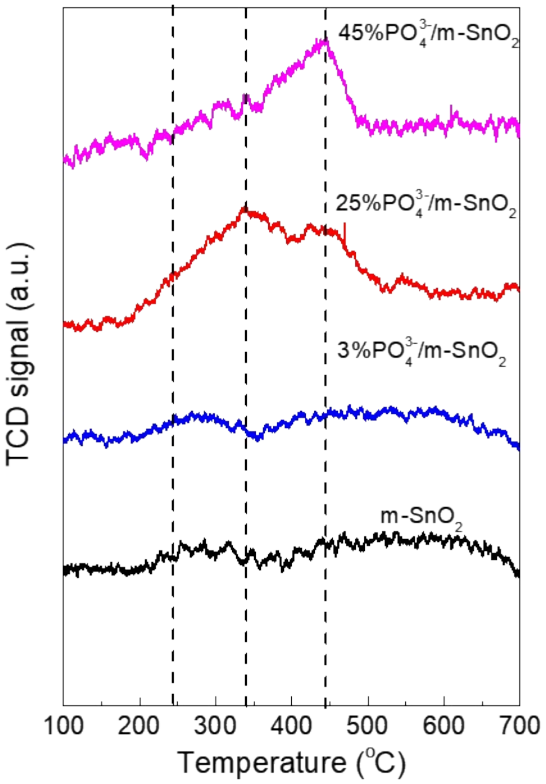

Figure 5S: NH_3-TPD profiles of the m-SnO_2, 3%PO_4^{3-}/m-SnO_2, 25%PO_4^{3-}/m-SnO_2 and 45%PO_4^{3-}/m-SnO_2 calcined at 400 ^{0}C

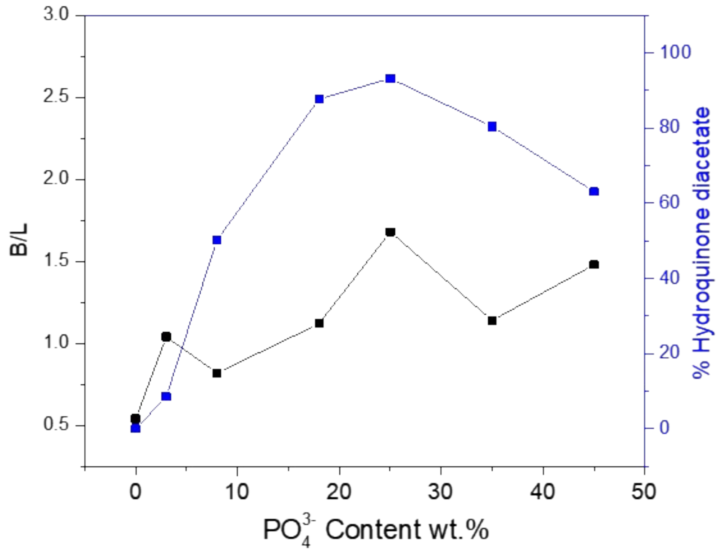

Figure 6S: Effect of PO_4^{3-} content wt.% /mSnO_2 calcined at 400 ^{0}C, on the ratio of Brönsted acid sites to Lewis acid sites and % hydroquinone diacetate.

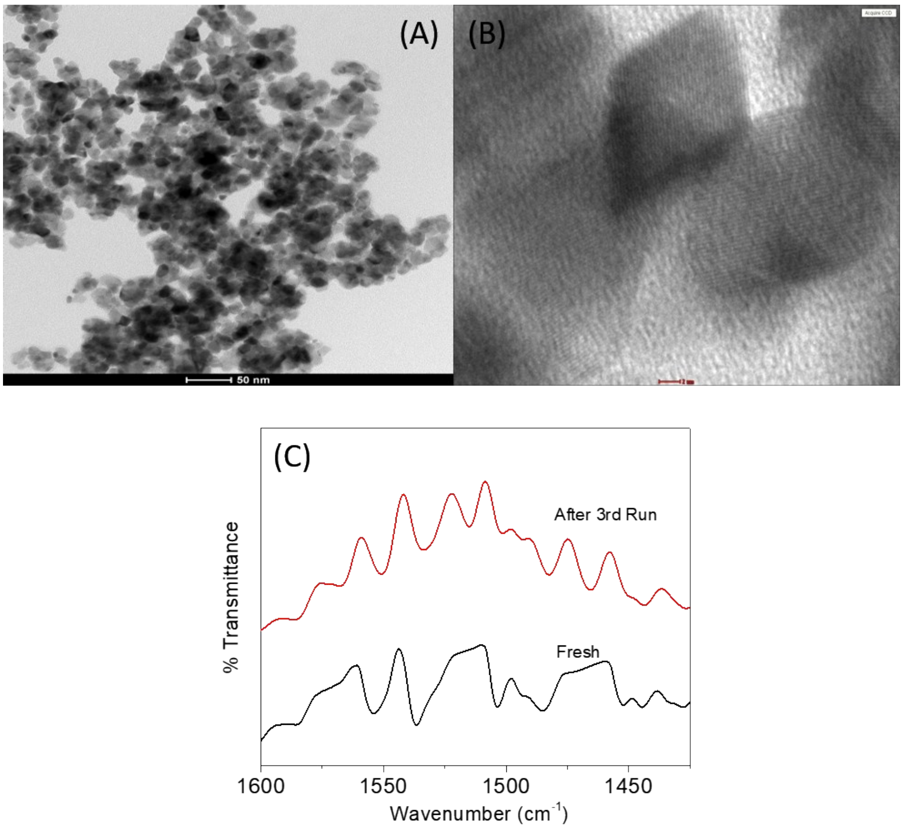

Figure 7S: (A) and (B) TEM images of 25% PO_4^{3-}/mSnO_2 calcined at 400 ^{0}C after 3rd run (C) FT-IR spectra of pyridine adsorbed on 25% PO_4^{3-}/mSnO_2 calcined at 400 ^{0}C after 3rd run

Table S1: comparison between solid acid catalyst and homogenous acid catalyst using 0.022 mmol of acid.

sample	Conversion %
25% $PO_4^{3-}/mSnO_2$	93.2
H_2SO_4	82.4
HCl	64.7

mmol of Acid for the 25%$PO_4^{3-}/mSnO_2$ has been calculated from the total number of acid sites. For the H_2SO_4 and HCl the same amount of acid concentration has been used
